# Characterisation of two quorum sensing systems in the endophytic *Serratia plymuthica *strain G3: differential control of motility and biofilm formation according to life-style

**DOI:** 10.1186/1471-2180-11-26

**Published:** 2011-02-01

**Authors:** Xiaoguang Liu, Jinli Jia, Roman Popat, Catherine A Ortori, Jun Li, Stephen P Diggle, Kexiang Gao, Miguel Cámara

**Affiliations:** 1 Institute of Life Sciences, Jiangsu University, Zhenjiang 212013, China; 2 School of Molecular Medical Sciences, Centre for Biomolecular Sciences, University of Nottingham, Nottingham NG7 2RD, UK; 3 School of Pharmacy, University of Nottingham, Nottingham NG7 3RD, UK; 4 Department of Plant Pathology, Shandong Agricultural University, Taian, China

## Abstract

**Background:**

*N*-acylhomoserine lactone (AHL)-based quorum sensing (QS) systems have been described in many plant-associated Gram-negative bacteria to control certain beneficial phenotypic traits, such as production of biocontrol factors and plant growth promotion. However, the role of AHL-mediated signalling in the endophytic strains of plant-associated *Serratia *is still poorly understood. An endophytic *Serratia *sp. G3 with biocontrol potential and high levels of AHL signal production was isolated from the stems of wheat and the role of QS in this isolate was determined.

**Results:**

Strain G3 classified as *Serratia plymuthica *based on 16S rRNA was subjected to phylogenetic analysis. Using primers to conserved sequences of *luxIR *homologues from the *Serratia *genus, *splIR *and *spsIR *from the chromosome of strain G3 were cloned and sequenced. AHL profiles from strain G3 and *Escherichia coli *DH5α expressing *splI *or *spsI *from recombinant plasmids were identified by liquid chromatography-tandem mass spectrometry. This revealed that the most abundant AHL signals produced by SplI in *E. coli *were *N*-3-oxo-hexanoylhomoserine lactone (3-oxo-C6-HSL), *N*-3-oxo-heptanoylhomoserine lactone (3-oxo-C7-HSL), *N*-3-hydroxy-hexanoylhomoserine lactone (3-hydroxy-C6-HSL), *N*-hexanoylhomoserine lactone (C6-HSL), and *N*-heptanoyl homoserine lactone (C7-HSL); whereas SpsI was primarily responsible for the synthesis of *N*-butyrylhomoserine lactone (C4-HSL) and *N*-pentanoylhomoserine lactone (C5-HSL). Furthermore, a quorum quenching analysis by heterologous expression of the *Bacillus *A24 AiiA lactonase in strain G3 enabled the identification of the AHL-regulated biocontrol-related traits. Depletion of AHLs with this lactonase resulted in altered adhesion and biofilm formation using a microtiter plate assay and flow cells coupled with confocal laser scanning microscopy respectively. This was different from the closely related *S. plymuthica *strains HRO-C48 and RVH1, where biofilm formation for both strains is AHL-independent. In addition, QS in G3 positively regulated antifungal activity, production of exoenzymes, but negatively regulated production of indol-3-acetic acid (IAA), which is in agreement with previous reports in strain HRO-C48. However, in contrast to HRO-C48, swimming motility was not controlled by AHL-mediated QS.

**Conclusions:**

This is the first report of the characterisation of two AHL-based quorum sensing systems in the same isolate of the genus *Serratia*. Our results show that the QS network is involved in the global regulation of biocontrol-related traits in the endophytic strain G3. However, although free-living and endophytic *S. plymuthica *share some conservation on QS phenotypic regulation, the control of motility and biofilm formation seems to be strain-specific and possible linked to the life-style of this organism.

## Background

Endophytic bacteria reside within the living tissue of their host plants without substantively harming it [1]. They can be beneficial to their host by promoting plant growth or acting as biocontrol agents [2,3]. *Serratia plymuthica *is ubiquitously distributed in nature, and most frequently associated with plants. This organism has been isolated from the rhizosphere and the phyllosphere of various plants, as an endophyte from the endorhiza of potato [4,5], or as a contaminant in a raw vegetable processing line [6,7]. Over the last two decades, *S. plymuthica *has received steadily increasing attention as a biocontrol agent for mainly fungal diseases. Based on the international approved German directive (TRBA 466), it is nowadays classified within the risk group 1 by the DSMZ (German Collection of Micro-organisms and Cell Cultures), indicating that the species does not pose a threat to human health [5].

Quorum-sensing (QS) plays a central role within a number of bacterial gene regulatory networks by controlling gene expression in a population-dependent manner with the aid of small diffusible signal molecules [8]. In Gram-negative bacteria, *N*-acylhomoserine lactones (AHLs) are the best described QS signal molecules. AHLs are made by LuxI homologues and, when they reach a critical threshold concentration, activate their cognate LuxR-type regulators which in turns induce or repress multiple gene expression. QS systems are involved in various physiological processes in bacteria, including bioluminescence, conjugation, symbiosis, virulence and biofilm formation [9]. Biofilms are increasingly recognized as the predominant form of bacterial growth in the environment [10]. Growth in a biofilm provides many advantages for bacteria, including enhanced resistance to environmental stresses, such as desiccation and antimicrobials, as well as to host defenses [11]. It has been well documented that a number of plant beneficial rhizobacteria employ AHLs as signal molecules to regulate biocontrol activities including the triggering of systemic resistance in host plants and the production of antifungal compounds [12-15]. The phenotypes regulated by AHLs in *Serratia *species are remarkably diverse and of profound biological and ecological significance. These include motility and biofilm formation, production of antibiotics, exoenzymes and butanediol fermentation, synthesis of the plant growth promoting auxin indole-3-acetic acid (IAA) and promotion of plant colonisation and biocontrol against several plant diseases [13-16]. However, the role of AHL-mediated QS system(s) in the endophytic strains of plant associated *Serratia *is less well understood.

Quorum quenching can been achieved by degrading AHL signal compounds using lactonases or acylases and has been shown to interfere with expression of AHL-regulated traits, such as virulence in *Erwinia carotovora*, *Erwinia amylovora *[17,18], *Burkholderia thailandensis *[19], *Burkholderia cepacia *complex [20], and *Pseudomonas aeruginosa *[21,22]; or biocontrol activity in rhizospheric *Serratia plymuthica *HRO-C48 [14]. AiiA-dependent signal degradation is a particularly useful tool to study the impact of quorum sensing in Gram-negative bacteria having multiple AHL regulatory circuits without the need to make mutants in the different AHL synthase genes [21].

In this study we describe the initial characterisation of two AHL-mediated QS systems in the wheat stem endophyte *Serratia plymuthica *G3 [23]. Two *luxIR *homologue genes, *splIR *and *spsIR *were identified from this strain, their AHL profiles characterised and their role in biocontrol traits were determined. The results presented show that whilst the control of some biocontrol traits by AHLs is conserved in distinct *S. plymuthica *isolates, the regulation of motility and biofilm formation is strain specific and possibly linked to the original environment of the isolate. These results provide new insights into the regulation of beneficial interactions between endophytic *Serratia *strains, pathogens and host plants and will help with the understanding of the inconsistencies in their biocontrol performance.

## Methods

### Microorganisms, media and growth conditions

The bacterial, fungal strains and plasmids used in this study are listed in Table 1. *S. plymuthica *G3 was isolated from the stems of wheat (*Triticum aestivum *L.) in Taian, Shandong, China. A spontaneous mutant resistant to rifampicin was selected for further experiments. *S. plymuthica *G3, its derivatives and the biosensor *Chromobacterium violaceum *CV026 [24] were grown in LB medium at 28°C and stored at -80°C in 25% glycerol. When required, antibiotics were added at final concentrations of 100 μg/ml for ampicillin, 100 μg/ml for carbenicillin, 40 μg/ml for rifampicin, and 25 μg/ml for tetracycline. All antibiotics were purchased from Sigma. The fungal isolate *Cryphonectria parasitica *was from the authors' laboratory collection and was routinely cultured on potato dextrose agar (PDA) (Difco) at 25°C.

**Table 1:** Bacterial strains and plasmids used in this study

Strain/Plasmid	Description	Reference/source
**Bacterial strain**		
*Serratia *sp. G3	Wild type, Rif ^r^	This work
G3/pME6000	G3 derivative transformed with the pME6000 vector plasmid	This work
G3/pME6863-aiiA	G3 derivative transformed with the pME6863 plasmid	This work
*Chromobacterium violaceum *CV026	Violacein production-based AHL bioreporter	24
*E*. *coli *DH5α	F- *recA1 endA1 hsdR17 deoR thi-1 supE44 gyrA96 relA1 *D(*lacZYA ± argF*) *U169 k*- [*u*80d*lacZ*DM15]	25
*E*. *coli *S17-1	*thi pro hsdR recA*; chromosomal RP4; Tra^+^; Sm/Sp^r^	25
**Plasmid**		
pME6000	Broad-host-range cloning vector; Tc^r^	21
pME6863	pME6000 carrying the *aiiA *gene of strain A24 under the control of constitutive *lac *promoter; Tc^r^	21
pUCP18::gfpmut3.1	pUCP18 carrying *gfpmut*3.1 gene; Cb^r^	28
**Fungal strain **		
*Cryphonectria parasítica*		Lab collection

### DNA preparation and manipulations

Standard methods were used for plasmid and genomic DNA isolation, restriction enzyme digestion, agarose gel electrophoresis, ligation, and transformation [25].

### Phylogenetic analysis

To gain a better taxonomic understanding of the *Serratia *G3 isolate a 16S rDNA-based phylogenetic tree was compiled using the neighbour-joining method of MEGA 4. The 16S rRNA gene sequence from the G3 isolate, we recently published elsewhere [23] was analysed together with those from other members of the genus *Serratia*, including the *S. plymuthica *DSM 4540 type strain as a reference and the related strains *S. proteamaculans *DSM 4543, *S. ficaria *DSM 4569, *S. entomophila *DSM 12358, *S. odorifera *DSM 4582, *S. marcescens *DSM 30121, as well as *S. plymuthica *RVH1 from a raw vegetable processing line and an endophytic strain JA05 isolated from ginseng plants. In addition, *Escherichia coli *ATCC 25922 as an outgroup. These 16S rRNA sequences were obtained from GenBank. The tree topology was tested by bootstrap analysis of 1000 samplings.

### Cloning and sequencing of two pairs of LuxIR homologues from *S. plymuthica *strain G3

Production of AHL signal molecules in strain G3 was detected using a T-streak assay with *C. violaceum *CV026 on plates. The following two pairs of primers for the cloning the *splIR *and *spsRI *loci were designed to the conserved regions of the corresponding genes in the genus *Serratia *using the ClustalW multiple sequence alignment program: SplIR-F: 5'-TTTGTAGAATACCGGCAAGCTGTT -3' and SplIR-R: 5'-CAGATCGTCACGGAGCCTGT-3'; SpsRI-F:5'-GAGAGGGTTCAGTGTCAAAT-3' and SpsRI-R: 5'-CCATGGAAGATGTAGAAATG-3'. These genes were amplified using G3 genomic DNA as a template by PCR and cloned into pMD-19T (Takara, Dalian, China). The clones expressed the AHL synthases SplI or SpsI in *E. coli *DH5α were selected by T-streak with *C. violaceum *CV026 for further identification of AHL profiles, and confirmed by PCR and sequencing (Sangon Co. Ltd., Shanghai, China). A neighbour-joining tree of LuxI family members was produced using the MEGA 4. Amino acid sequences of SplI and SpsI from the G3 isolate were aligned and analysed together with LuxI homologs from other eight members of *Serratia *and EsaI from *Pantoea stewartii *DC283. TraI of *Agrobacterium vitis *S4 was tested as outgroup. These amino acid sequences of LuxI homologs were obtained from GenBank. Confidence in neighbour-joining tree was determined by analysing 1000 bootstrap replicates.

### AHL degradation by heterologous expression of the AiiA acyl-homoserine lactonase

A quorum-quenching approach was used to identify AHL-regulated biocontrol-related phenotypes in the endophytic strain G3. *E. coli *S17-1/pME6863 carrying the AHL-lactonase *aiiA *from the *Bacillus *sp. strain A24 under the control of the constitutive *lac *promotor [21] was used to mobilise *aiiA *into G3 by conjugation to obtain G3/pME6863-*aiiA*. G3 containing pME6000 was used as a control. Transconjugants were selected on LB plates containing 25 μg/ml of tetracycline and 40 μg/ml of rifampicin. Inactivation of the AHLs produced by strain G3 was evaluated by T-streak with the *C. violaceum *CV026 biosensor strain and further confirmed by LC-MS/MS analysis as described below.

### Extraction of AHLs from culture supernatants

For extraction of signal molecules, all tested bacteria were grown in 10 ml of LB overnight at 28°C with shaking. Cell-free culture supernatants (sterilized by passing through a 0.2-μm pore filter) were extracted twice with equal volumes of ethyl acetate after which the extracted organic phases were pooled. The solvent was removed under vacuum and the resulting extract reconstituted in acetonitrile prior to LC-MS/MS analysis.

### Identification of AHL profiles by LC-MS/MS

AHLs were examined by LC-MS/MS in the Centre for Analytical Bioscience, School of Pharmacy, University of Nottingham, UK. Briefly, the mobile phase A (Aqueous) was 0.1% formic acid in water (Sigma, MS grade) and mobile phase B (Organic) 0.1% formic acid in acetonitrile (Fisher). Two Shimadzu LC-10ADvp pumps in binary mode were run at 0.45 ml/min using the gradients as follows: isocratic flow at 0% for 1 min, linear gradient from 0 to 50%B in 1.5 min, 70 to 99% until 5.5 min, isocratic until 7.5 min. The column was re-equilibrated for a further 4 min including subsequent injection cycle time. The autosampler was a Shimadzu SIL-HTc. The column, a Phenomenex Gemini C18 (5 u) 3 × 15 mm was held at 50°C in a Shimadzu oven, model CTO-10Avp. The MS detector was a 4000 QTrap from Applied Biosytems. Specific analyses were monitored in a targeted multi-reaction monitoring (MRM) mode in which all specific source and collision cell parameters had been optimized. Generic parameters were: ion source voltage 5000 V, source temperature 450°C, the curtain, collision activated dissociation gas (CAD, N_2_), nebulizer gas (GS1) and heater gas (GS2) set at 20, 6, 30 and 15 psi respectively. The quadrupoles were set at unit resolution and specific precursor-product ion pair parameters were determined automatically using the quantitative optimization facility of Analyst 1.4.1. Subsequent ion trap scans (enhanced product ion, EPI) were triggered by ion counts in any one MRM channel rising above 5000 counts per scan (cps). During these EPI scans, the declustering potential was ramped from 15 to 35 V and the collision energy was ramped between 20 and 80 V. Product ions were monitored in the range 80 to 330, with a default fill time of 250 msec using dynamic fill time and a scan rate of 1000Th/sec. Relative quantification was performed by peak integration of the extracted ion chromatogram of the relevant MRM ion channel. The LC/MS system was controlled by the Analyst 1.4.1 software and data analysis was performed using the same in quantitative mode. A wide selection of synthetic AHLs with or without a 3-oxo or 3-hydroxy substitution and with acyl side-chain lengths ranging from C4 to C14 each at either 1 μM or 5 μM concentrations, for AHLs with even and odd carbon chain lengths respectively, were used as standards. AHLs were identified and confirmed by comparing both the elution time and the MS spectra of the peaks obtained with those of the standards.

### Antifungal activity *in vitro*

The antagonistic activity of G3 and its derivatives G3/pME6863-*aiiA *and G3/pME6000 were tested against the phytopathogenic fungus *Cryphonectria parasitica*, the causal agent of chestnut blight as previously described [13].

### Motility assays

Minimal swim motility agar plates contained 10 g/liter tryptone, 5 g/liter NaCl and 0.3% (wt/vol) Bacto agar [26]. A 1 μl volume of overnight seed cultures grown at 28°C were inoculated onto swim agar plates and incubated at 28°C for 16 h.

### Adhesion assays

Adhesion is considered to be the first step in the development of bacterial biofilm. Bacterial adhesion on abiotic surface was measured using polystyrene microtitre plates in triplicate as described by O'Toole and Kolter, 1998 [27] with a few modifications. Overnight bacterial cultures were inoculated into the wells of microtiter plates in 100 μl of LB or M9 medium (final concentration of OD_600 _0.02) without shaking and incubated at 30°C for 24, 48 and 72 h, respectively. At 24 h intervals, the cell densities were determined at 600 nm, followed by quantification of adhesion. The medium was removed, and the cells were stained with 0.1% solution of crystal violet (CV) at room temperature for 20 min. The dye was then removed and the wells were washed four times. Bound dye CV was solubilized with 95% ethanol, and the absorbance was measured at 570 nm.

### Flow cell biofilm assays

Firstly the strains G3/pME6863-*aiiA *and the vector control G3/pME6000 were tagged with the green fluorescent protein, GFP by electroporation with plasmid pUCP18::gfpmut3.1 [28]. The transconjugants were selected on LB plates supplemented with both tetracycline and carbenicillin, and verified through observation under the fluorescence microscope. Biofilms were cultivated in a modified flow chamber in ×20 diluted LB. 100 μl of bacterial overnight cultures (OD_600 _= 0.1) were injected into each channel of flow cell and incubated at room temperature for 48 hours, at flow rate of 52.04 μl/ml for each channel.

### Capturing of confocal images

Biofilms were visualized with an inverted Zeiss LSM700 microscope. The objective used was a Zeiss EC Plan-Neofluar 10x/0.30. 6 replicate Z-Stacks, with an interval of 5.741 μm and the pinhole at 1AU, were acquired from each flow cell and used to create three-dimensional representations of the biofilms. Biofilm structure was quantified from the Z stacks using the image analysis software package COMSTAT [29].

### Production of exoenzymes, siderophores and indole-3-acetic acid (IAA)

Proteolytic and chitinolytic activities and siderophores production were assayed as described previously [30,31]. HPLC (Agilent 1200LC) analysis of IAA production was performed as previously described [23,32].

### Statistical analysis

All data were subjected to analysis of variance (ANOVA) using Fisher's least significant difference (LSD) and Duncan's multiple-range test to compare treatment mean values. Each trial was repeated at least twice with at least three replicates for each treatment.

### Nucleotide sequence accession numbers

The GenBank accession numbers for the *splIR*, and *spsRI *genes from strain G3 are FJ919305 and FJ919306, respectively.

## Results

### Phylogenetic classification of *S. plymuthica *G3

To classify phylogenetically the G3 strain isolated from wheat stems, the sequence from the 1474-bp fragment of 16S rDNA from this isolate we previously determined (EU344964) [23] was subjected to phylogenetic analysis with different 16S rDNA sequences from members of the genus *Serratia *and *E. coli *strain ATCC 25922 as the outgroup. The sequence alignment for the phylogenetic tree was constructed and evaluated with MEGA 4 using the neighbour-joining method (see Additional file 1). As the phylogenetic tree showed that the G3 isolate was clustered within the same group (confidence = 99%) with RVH1 (AY394724) and the type strain DSM 4540 (AJ233433) of *S. plymuthica*, respectively. Therefore, *Serratia *sp. G3 was tentatively classified as *S. plymuthica*. It is worth noting that the atypical *S. plymuthic*a RVH1 strain is unable to produce prodigiosin pigment when compared to the *S. plymuthica *DSM 4540 type strain, but a combined comparative analysis of 16S rRNA and *gyrB *sequences, DNA-DNA hybridization, and biochemical characteristics unequivocally identified this strain as *S. plymuthica *[7].

### *S. plymuthica *G3 possesses two quorum sensing systems SplIR and SpsRI

The homologues of the two LuxIR genes were cloned from strain G3 by PCR using primers against conserved sequences as described in the Material and Methods. PCR products of 1441-bp and 1391-bp corresponding to the expected *splIR *and *spsIR *respectively were sequenced. The 1441-bp resulting sequence included two open reading frames (ORFs) corresponding to the predicted AHL synthase gene *splI *(633-bp) and the response regulator gene *splR *(750-bp) (FJ919305). The *splI *and *splR *ORFs are convergent, overlapping by 29-bp in their 3' regions. The 1391-bp sequenced fragment carried two ORFs corresponding to the predicted AHL synthase gene *spsI *(687-bp) and the response regulator gene (*spsR*) (747-bp) (FJ919306). The *spsR *and *spsI *ORFs are also convergent and overlapping by 54-bp in their 3' regions.

**Figure 1:**
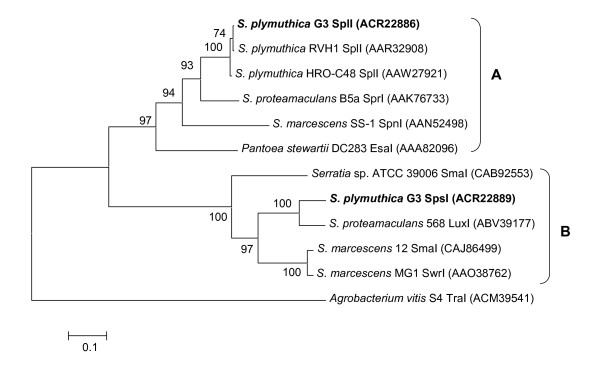
**Neighbour-joining tree of LuxI family members in *Serratia***. The phylogenetic tree was generated using MEGA 4. LuxI family members in *Serratia *are clustered into two groups according to the AHL patterns. SplI and SpsI from G3 were in group A and group B, respectively. The significance of each branch is bootstrap value calculated for 1000 subsets. Scale bar indicates the mean number of substitutions per site.

Database searches using tblastx revealed that SplI (ACR22886) shares 99% and 98% identity to SplI (AAR32908, AAW27921) from *S. plymuthica *strains RVH1 and HRO-C48, respectively, as well as 83, 68, 67% identity to the SprI (AAK76733) from *Serratia proteamaculans *B5a, SpnI (AAN52498) from *S. marcescens *SS-1, and EsaI (AAA82096) from *Pantoea stewartii *DC283 respectively, which are mainly responsible for the synthesis of 3-oxo-C6-HSL [15,16,33-36]. The second LuxI homolog SpsI from G3 was most similar to the LuxI homolog (ABV39177) from the poplar endophytic bacterium *S. proteamaculans *568 (86% identity and 92% similarity), then to SwrI (AAO38762) from *Serratia marcescens *MG1 (74% identity) and to SmaI (CAJ86499) from *S. marcescens *strain 12 (67% identity), SmaI (CAB92553) from *Serratia *strain ATCC 39006 (60% identity). The AHL synthases SwrI and SmaI catalyze preferentially the synthesis of C4-HSL and, in less amount, C6-HSL [16,37,38]. To examine the evolutionary relationship between the LuxI family members described above, a phylogenetic analysis was performed using MEGA 4 and the neighbour-joining tree was showed in Figure 1. The results were consistent with the similarity analysis of amino acid sequences within LuxI family members, the LuxI family synthases were clustered into two groups, and SplI and SpsI from strain G3 are classified into group A and group B, respectively.

### SplI and SpsI from *S. plymuthica *G3 produce multiple AHLs

To determine which AHLs were made by each SplI and SpsI, LC-MS/MS analysis was performed on extracted culture supernatants from the wild type G3 strain as well as recombinant *E. coli *strains expressing *splI *or *spsI *and the spectra profiles compared to that of synthetic AHL standards. At least ten different AHLs were detected in varying abundance in the wild type G3, including unsubstituted AHLs (C4-HSL, C5-HSL, C6-HSL, C7-HSL, C8-HSL), 3-oxo derivatives (3-oxo-C6-HSL, 3-oxo-C7-HSL, 3-oxo-C8-HSL) and 3-hydroxy derivatives (3-hydroxy-C6-HSL, 3-hydroxy-C8-HSL). The most abundant and hence most likely biologically relevant AHLs detected in the spent culture supernatants of the endophytic strain G3 were 3-oxo-C6-HSL, C4-HSL, C6-HSL, 3-hydroxy-C6-HSL and 3-oxo-C7-HSL. However, strain G3 did not produce long chain AHLs [23]. When expressed in *E. coli *(Table 2), the recombinant SplI produced all ten AHLs whereas SpsI produced only unsubstituted AHLs, including C4-HSL, C5-HSL, C6-HSL, C7-HSL, and C8-HSL. The most abundant one was C4-HSL from SpsI, 100 fold higher than that the production of this molecule by SplI in *E. coli*, suggesting that SpsI is could also be the main AHL synthase responsible for synthesis of this AHL in G3, in accordance with SwrI and SmaI from different *S. marcescens *strains [37,38] which share similarity to SpsI. Both SpsI and SplI produce C6-HSL, but only SplI was responsible for the most abundant signal 3-oxo-C6-HSL, that is similar to SplI from *S. plymuthica *strains HRO-C48 and RVH1 [14,32], SprI from *S. proteamaculans *B5a, SpnI from *S. marcescens *SS-1 [34,35], as well as EsaI from *P. stewartii *[36]. However, 3-oxo-C7-HSL and 3-hydroxy-C6-HSL have not been reported as the main AHL signals in other members of *Serratia *with the exception of *S. plymuthica *IC1270 which showed very weak production of the predicted 3-hydroxy-C6-HSL by TLC analysis [30]. It is worth noting that there might be differences between AHL ratios from SplI and SpsI expressed in the wild type G3 and *E. coli*.

**Table 2:** AHL production by *E. coli *expressing either *splI *or *spsI *from G3

**AHL produced by G3 WT**[23]	**AHL expressed in *E. coli/*splI**^**#**^	**AHL expressed in *E. coli*/spsI**^**#**^
C4-HSL	+	++++
C5-HSL	+	+++
C6-HSL	++	++
C7-HSL	++	+
C8-HSL	+	+
3-oxo-C6-HSL	+++	-
3-oxo-C7-HSL	++	-
3-oxo-C8-HSL	+	-
3-hydroxy-C6-HSL	++	-
3-hydroxy-C8-HSL	+	-

AHL profiles identification was performed by LC-MS/MS from two independent experiments.

^# ^AHL mass abundance (relative quantity of íons from a particular AHL relative to that of a known standard) on LC-MS/MS: ++++ indicates 10^7^; +++ indicates 10^6^; ++ indicates 10^5^; + indicates ≤10^4^.

### Heterologous expression of *aiiA *in G3 abolishes AHL accumulation and has an impact on biocontrol traits

A number of bacteria are known to regulate various cell processes, including biocontrol activities through AHL-mediated quorum sensing systems. To determine the ability of the *Bacillus *A24 lactonase AiiA in degrading AHL signal molecules in G3, the plasmid pME6863-*aiiA*, and the control vector pME6000 (lacking the *aiiA *gene) were introduced into the wild type G3 by mating with the *E. coli *donor strain S17-1. Overnight culture supernatants from these transconjugants were extracted in duplicate with solvent and subjected to LC-MS/MS semiquantitative analysis based on MRM mode showing that G3 harbouring the pME6000 vector control exhibited similar AHL patterns and concentration to the wild type (data not shown). In contrast, AHL production was practically abolished in G3 expressing *aiiA *from pME6863-*aiiA *(more than 99% reduction), with only trace amounts of C4-HSL remaining which could not be detected by the biosensor CV026 and hence were unlikely to influence gene expression. This result suggested that AiiA can efficiently degrade all series of AHLs, including unsubstituted, 3-oxo, and 3-hydroxy at the third carbon position as it has been previously shown [39].

Impairment in AHL accumulation resulted in down-regulation of the chitinolytic and proteolytic activities in G3/pME6863-*aiiA*. In contrast, biosynthesis of IAA increased five-fold and there was no effect on production of siderophores, compared to the wild type G3 and the control G3/pME6000 (see Additional file 2). This is in agreement with previous observations in *S. plymuthica *HRO-C48 heterologously expressing *aiiA *[14].

**Figure 2:**
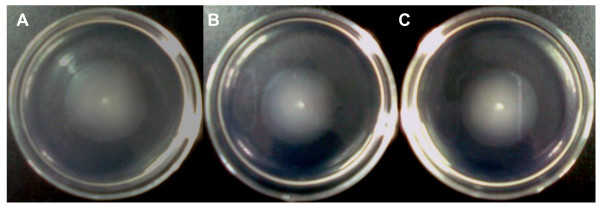
**Swimming motility by G3 is independent of AHL signalling**. One microlitre of overnight cultures of the wild type G3 (A), the control G3/pME6000 (B) and G3/pME6863-*aiiA *(C) were inoculated onto swim agar plates and incubated at 28°C for 16 h.

Swimming motility was also assayed to determine the role of quorum quenching by AiiA in motility. The swimming zones of the wild type G3, the AHL quenched strain G3/pME6863-*aiiA *and the vector control G3/pME6000 after incubation for 16 h at 28°C were 33.75 ± 0.75 mm, 33.08 ± 0.80 mm, and 32.83 ± 0.14 mm, respectively. The results suggest that, in contrast to *S. plymuthica *HRO-C48 where QS negatively regulates this phenotype [14], heterologous expression of *aiiA *in G3 has no influence on swimming motility (Figure 2).

### Lactonase expression in *S. plymuthica *G3 reduces antifungal activity *in vitro*

Strain G3 exhibited inhibitory effects against several phytopathogenic fungal isolates *in vitro *and *in vivo *(data not shown). To determine the effect of quorum quenching by lactonase on antifungal activity, dual cultures were carried out, on single PDA plates, of the strain G3, G3/pME6863-*aiiA *or G3/pME6000 with *C. parasitica*, the cause of chestnut blight. After incubation for 4 days at 25°C, the radius of the inhibition zones was measured. Although no large differences were observed between the wild type G3 and the control strain G3/pME6000, the radius of inhibition zones produced by G3/pME6863-*aiiA *was significantly decreased compared with the control G3/pME6000 and the wild type G3 at *P *= 0.01 for *C. parasitica *(Table 3.). The data showed that antifungal activity by G3 is partially dependent on AHL signaling via regulation of various exoenzymes and secondary metabolites.

**Table 3:** Effect of quorum quenching on antifungal activity *in vitro*

Phytopathogenic fungus	Inhibition zone (mm)*		
	
	G3 (wt)	G3/pME6863-*aiiA*	G3/pME6000
*Cryphonectria parasitica*^*a*^	8.25 ± 0.42 (A)	5.91 ± 0.20 (B)	8.33 ± 0.51 (A)

* Radius of inhibition zone on PDA plates in dual culture for 4 days, Data represents mean values ± SD with six replicates.

^a ^Different letters in the same line indicate significant differences at P < 0.01

### Abiotic surface adhesion and biofilm formation in *S. plymuthica *G3 are affected by lactonase expression

**Figure 3:**
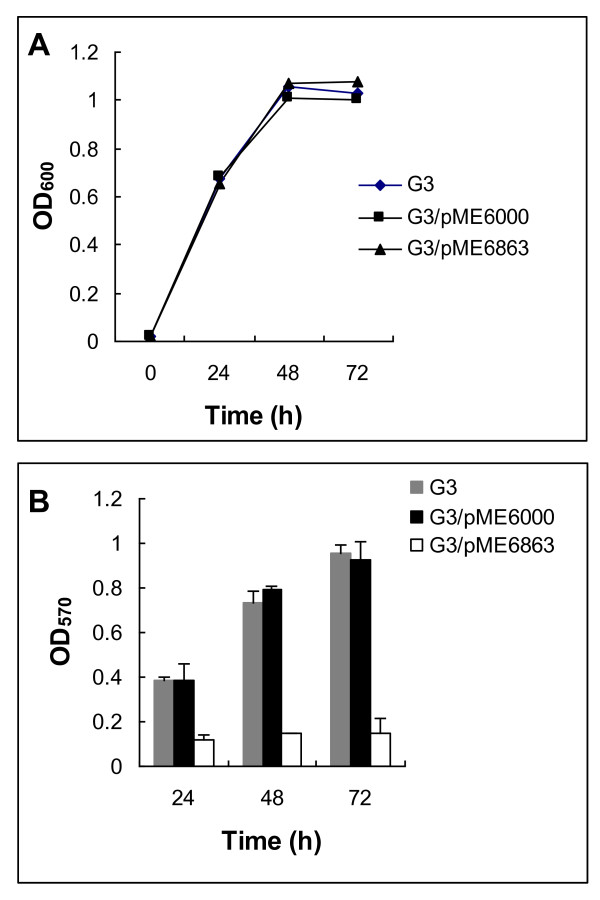
**Effect of *aiiA *expression on abiotic surface adhesion by *S. plymuthica *G3**. A: OD_600 _of G3 bacterial cultures in the presence and absence of the *aiiA *lactonase gene. B: Absorbance of crystal violet at 570 nm from stained cells bounds to polystyrene microtitre plate as a representation of adhesion. Experiments were done in triplicate.

Many bacteria rely on QS systems to govern various aspects of biofilm development, including adhesion, motility, maturation, and dispersion [10,37]. Using microtiter plate assays, we evaluated the impact of quorum quenching by *aiiA *on adhesion to abiotic surfaces in G3. Figure 3A illustrates by OD_600_, there are no significant difference in bacterial growth rate between the wild type G3, G3/pME6000 and G3/pME6863-*aiiA*, but the strain G3/pME6863-*aiiA *showed a significant reduction in adhesion, compared with the vector control strain G3/pME6000 and the wild type G3 (Figure 3B).

**Figure 4:**
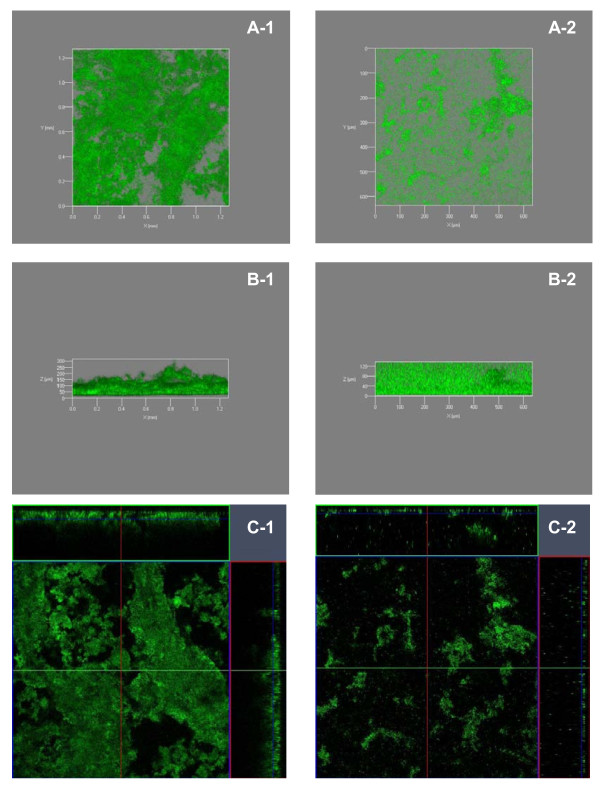
**Effect of quorum quenching on biofilm formation**. *In vitro *biofilm formation of the GFP-tagged strains G3/pME6000-pUCP18::gfpmut 3.1 (left panel) and G3/pME6863-pUCP18::gfpmut3.1 (right panel). Flow cell cultured biofilms incubated in 5% LB were observed by confocal laser scanning microscopy after 48 h. A: 2 dimensional optical slice and cross sections, B: 3 dimensional y-projection; C: 3 dimensional *z-*projection.

**Figure 5:**
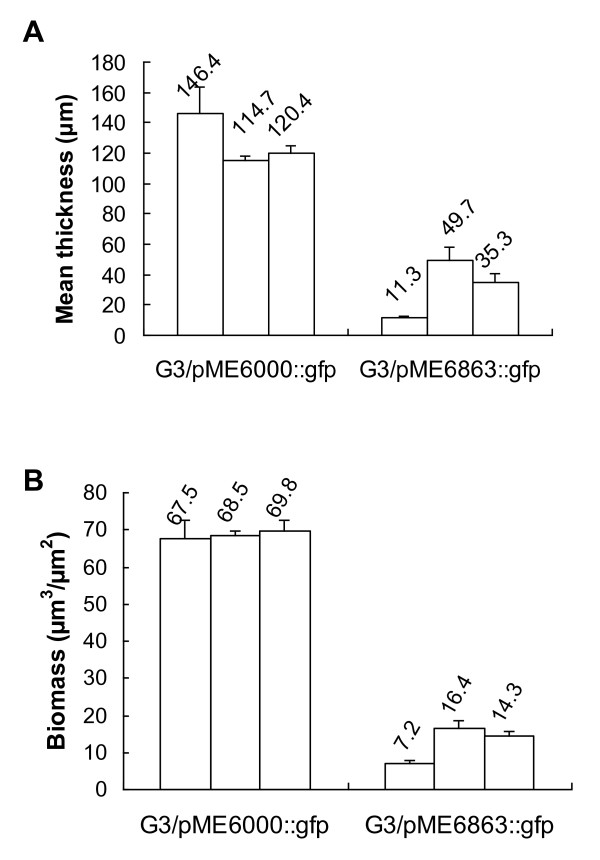
**Quantitative analysis of the impact of *aiiA *expression on biofilm formation**. The biofilm thickness (A) and the biomass (B) in flow cell were quantified by COMSTAT. Data represent mean ± standard error of 6 random measurements with three independent channels.

Furthermore, 48 hour flow cell cultures of GFP-tagged G3/pME6863-*aiiA *and G3/pME6000 were observed and quantified for biofilm formation using CLSM during two independent experiments. Figure 4A, B and 4C represent 2D optical slice and cross sections view (3D y and z-projection views) of bacterial biofilms developed after 24 h of growth by both strains. Quantitative analysis by COMSTAT indicated that not only the biofilm thickness (Figure 5A; the mean thickness of G3/pME6000::gfp and G3/pME6863::gfp biofilms is 127.17 ± 8.43 μm and 32.10 ± 5.10 μm respectively), but also the biomass (Figure 5B; the biomass of G3/pME6000::gfp and G3/pME6863::gfp biofilms is 68.62 ± 3.03 μm^3^/μm^2 ^and 12.63 ± 1.39 μm^3^/μm^2 ^respectively) between these two strains were significantly different, suggesting that biofilm development by G3, under the conditions used, is AHL-dependent.

## Discussion

Endophytic bacteria have been found in virtually every plant studied, and there is increasing interest in developing their biotechnological potential to improve phytoremediation and the sustainable production of non-food crops for biomass and biofuel production [3]. In this manuscript we have reported that a new isolate of endophytic *Serratia plymuthica *G3 from the stems of wheat, exhibiting antifungal activities, produces high levels of AHLs and that the QS control of swimming motility and biofilm formation shows significant differences to other isolates of this organism from different environments previously described.

The ability of *Serratia *strains to produce AHLs and their AHL production profiles is well known to be species- and strain-dependent [16]. Previous works have also demonstrated that in *S. marcescens *SS-1 and *S. plymuthica *strains RVH1 and HRO-C48, SpnI or SplI knock out mutations abolished the production of 3-oxo-C6-HSL completely, but still retained residual AHL signals, suggesting the presence of additional AHL synthase(s) in some species of *Serratia *[15,33,35]. However, this is the first report showing the identification and initial characterisation of two QS systems *splIR *and *spsIR *in a single *Serratia *isolate. Sequence analysis showed that SplIR is highly similar to the SplIR of *S. plymuthica *strains RVH1 and HRO-C48, as well as SprIR of *S. proteamaculans *B5a and *S. marcescens *SS-1, all of which are responsible for the biosynthesis of 3-oxo-C6-HSL, and C6-HSL. Whereas SpsIR shares similarity to SwrIR and SmaIR from *S. marcescens *MG1 and strain 12 respectively, which are responsible for production of unsubstituted C4-HSL and smaller amounts of unsubstituted C6-HSL and C8-HSL (Table 2). In addition, these two sets of *luxI *and *luxR *homologous genes organized convergently in *S. plymuthica *G3 chromosome is characteristic of the most γ-proteobacteria [33,35,40]. The results were in line with the phylogenetic analysis (Figure 1), demonstrating that the LuxI family members from the genus of *Serratia *can be clustered into groups A and B according to the main AHL signals produced by bacteria, but it is not species-specific. For example, *S. marcescens *SS-1 was classified into group A as SplI of G3, known to produce 3-oxo-C6-HSL. In contrast, Strain 12 and MG1 of *S. marcescens *were clustered into group B due to the production of C4-HSL as was SpsI from G3. Hence, our data provide new evidence to support that AHL patterns in *Serratia *is strain-dependent, indicating the presence of some conserved protein structure-function characteristics that would determine this specificity and which would be worth investigating in future. In addition, horizontal transfer of QS systems due to transposition or phage-mediated events have been described for the *spnIR *locus of *S. marcescens *SS-1 and the *smaIR *locus from strain 12 to 274 [16,38,41]. Consequently, the presence of two QS systems in G3 may have originated from horizontal gene transfer amongst members of the genus *Serratia*. Gray and Garey (2001) also deduced that multiple LuxI and/or LuxR homologues present within single species have been usually acquired from independent sources [40].

Further comparative analysis of AHL profiles using LC-MS/MS from the wild type G3 and *E. coli *DH5α expressing the recombinant plasmid carrying and *splI *or *spsI *showed that SplI is responsible for the synthesis of a broad range of AHLs with different substitutions whereas SpsI only drives the synthesis of AHLs with no substitutions on their acyl chains all of which are also made by SplI although some of them at much lower levels such as C4-HSL and C5-HSL. To our knowledge, the strain G3 is the only *Serratia *so far described with the ability to produce 3 different families of AHLs according to substitutions in position 3 (none, 3-oxo and 3-hydroxy), although this can be due to the improved LC-MS/MS techniques used with higher sensitivity to detect lower concentration and broader range of AHL signals. The most abundant AHL signals identified by LC-MS/MS from G3 were 3-oxo-C6-HSL and C4-HSL although significant levels of C6-HSL, 3-oxo-C7-HSL and 3-hydroxy-C6-HSL were also detected [23]. However, the individual biological role of these AHLs remains unknown. Overlaps between the AHL profiles produced by different LuxI homologues in a single organism has been previously described in other bacteria such as *Yersinia pseudotuberculosis *[42] and this usually results in very complex QS regulatory cascades with a tight intraregulation between them [43]. Whether this level of complexity exists in the G3 strain remains to be elucidated.

In many plant beneficial rhizobacteria, QS mechanisms induce the synthesis of antimicrobial secondary metabolites and extracellular lytic enzymes with inhibitory effects towards other bacteria, fungi, protozoa, and nematodes [12]. The quorum quenching strategy using the lactonase AiiA was exploited to simultaneously quench the two AHL systems discovered in the endophytic strain G3 of *S. plymuthica *and investigate their role in controlling biocontrol-related phenotypes. The phenotypic analysis revealed that the strain G3/pME6863 expressing *aiiA *had reduced antifungal activity, chitinolytic and proteolytic activities, but increased of IAA biosynthesis, and had no impact on siderophore production compared with the strain carrying the vector control G3/pME6000 and the wild type G3, indicating that QS control multiple biocontrol-related phenotypes in this strain. These results are in agreement with previous observations in the rhizospheric *S. plymuthica *HRO-C48 expressing AHL lactonases [14]. Depletion of AHLs with this lactonase resulted in altered adhesion and biofilm formation *in vitro*. This was different from the closely related *S. plymuthica *strains HRO-C48 and RVH1, where biofilm formation for both strains is AHL-independent. In addition, in contrast to HRO-C48, swimming motility was not controlled by AHL-mediated QS [14,33].

Attachment is required for biofilm formation and these are key processes in the interaction between bacteria and plant tissues which have been shown to rely on quorum sensing [44]. For example, in the biocontrol bacterium *Pseudomonas chlororaphis *strain 30-84, QS systems and their control over phenazine production play a role in the successful formation of surface-attached populations required for biofilm formation. Transcriptome analysis revealed that phenazines as signals, up-regulated many of the genes related to cell adhesion and biofilm development, such as fimbrial and lipopolysaccharides (LPS) genes [45]. The SwrIR quorum sensing system in *S. marcescens *MG1 plays a key role in biofilm development, from attachment to swarming motility, biofilm maturation and detachment, although QS regulation of adhesion in MG1 is surface dependent [37]. In *S. marcescens *strain 12, biofilm formation seems to rely on *smaI*, although this was measured using an attachment assay to a plastic microtitre plate [38], where SmaI is mainly responsible for C4-HSL synthesis. *Pantoea stewartii *causing Stewart's vascular wilt and leaf blight in sweet corn and maize utilizes the EsaI/EsaR QS system to control virulence and effective colonization. EsaI shares 80% similarity to SplI of G3 and is a typical AHL synthase that also catalyzes preferentially the synthesis of 3-oxo-C6-HSL. Both EsaI and EsaR mutants were not able to cycle through normal programmed bacterial development leading to the formation of atypical biofilms *in vitro *and loss of dissemination and infectivity *in vivo*, although the AHL-deficient strain displayed an unusually robust adhesion phenotype [36]. Under the experimental conditions used the ability of abiotic surface adhesion and biofilm formation by G3, using microtiter plate and flow cell assays respectively, is AHL-dependent, as the strain G3/pME6863 expressed *aiiA *was impaired in these phenotypes *in vitro*. In contrast, previous studies based on microtitre plate assays reported that biofilm formation by the closely related *S. plymuthica *strains HRO-C48 and RVH1 were not affected by AHL signalling. This was demonstrated by the heterologous expression AiiA or the use of a *splI*-mutant in which 3-oxo-C6-HSL production was abolished, but still retained residual unsubstituted AHLs [14,46]. This suggests that QS may have different roles in *S. plymuthica *isolates from different environments.

A number of different factors might affect adhesion, including physicochemical interactions between the bacterium and the substratum, flagella, fimbriae, outer membrane proteins, and the presence of extracellular polymers. For instance, quorum-sensing regulation of adhesion, biofilm formation, and sloughing in *S. marcescens *MG1 has been shown to be surface dependent, and under the control of nutrient cues [10,37]. We predict that the variations on QS regulation of biofilm development among different strains of *S. plymuthica *is likely to be influenced by strain-specificity or their life style though this remains to be further investigated.

Consequently, this study reveals that, in *S. plymuthica *G3, QS positively controls antifungal activity, production of exoenzymes, but negatively regulated production of indol-3-acetic acid (IAA). This is in agreement with previous reports in strain HRO-C48. However, in contrast to *S. plymuthica *strains HRO-C48 and RVH1, where biofilm formation is AHL-independent, in G3 adhesion and biofilm formation is controlled by QS. Finally, in contrast to HRO-C48, swimming motility is not under QS control in G3 [14-16,33]. This work indicates the existence of a differential role for QS between endophytic and free living bacterial isolates suggesting that this regulatory mechanism can evolve to maximise the adaptation to different lifestyles.

## Conclusions

Two QS systems SplIR and SpsIR from the endophytic *S. plymuthica *strain G3 have been characterised and their AHL profiles determined. This QS network is involved in global regulation of biocontrol-related traits, especially antifungal activity, adhesion and biofilm formation some of which are strain-specific in the Genus of *Serratia*. Further investigation will focus on the interplay between the two QS systems in strain G3 and the integration of QS into complex regulatory networks to modulate the beneficial plant-microbe interaction. This will ultimately lead to the optimisation of seed inoculums and provide novel strategies to improve the efficacy of *S. plymuthica*-mediated biocontrol and plant growth promotion.

## Abbreviations

AHL: N-acyl homoserine lactone; C4-HSL: *N*-butyrylhomoserine lactone; C5-HSL: *N*- pentanoylhomoserine lactone; C6-HSL: *N*-hexanoylhomoserine lactone; C7-HSL: *N*-heptanoylhomoserine lactone; C8-HSL: *N*-octanoylhomoserine lactone; 3-oxo-C6-HSL: *N*-3-oxo-hexanoylhomoserine lactone; 3-oxo-C7-HSL: *N*-3-oxo-heptanoyl homoserine lactone; 3-oxo-C8-HSL: *N*-3-oxo-octanoylhomoserine lactone; 3-hydroxy-C6-HSL: *N*-3-hydroxy-hexanoylhomoserine lactone; 3-hydroxy-C8-HSL: *N*-3- hydroxy-octanoylhomoserine lactone; BCA: Biocontrol agent; HPLC: High performance liquid chromatography; IAA: Indole-3-acetic acid; LC-MS/MS: Liquid chromatography-tandem mass spectrometry; QS: Quorum sensing;

## Authors' contributions

XL conceived and designed the study, carried out phylogenetic analysis, obtained GFP-tagged strains for biofilm analysis and wrote the paper; JJ, constructed strains of G3/pME6863-*aiiA *and G3/pME6000, and performed quorum quenching analysis of biocontrol phenotypes; RP, carried out flow cell biofilm assays and CLSM observation and images capture and statistical analysis; OCA, contributed to the LC-MS/MS identification of AHL profiles; JL, contributed to the AHL extracts and biofilm analysis; SPD, supervised biofilm assays and revised the manuscript; KG, contributed to the analysis of IAA and siderophores; MC, conceived and designed the study and critically revised the manuscript. All authors read and approved the final manuscript.

## Supplementary Material

Additional file 1 **Phylogenetic analysis of the 16S rRNA gene sequences**. The phylogenetic tree was produced using the 16S rRNA gene sequences corresponding to the endophytic strain G3 and other members of the genus *Serratia. Escherichia coli *ATCC 25922 was used as outgroup by the neighbour-joining method of MEGA 4. The significance of each branch is bootstrap value calculated for 1000 subsets. Scale bar indicates the mean number of substitutions per site. *^T ^Type strain. Click here for file

Additional file 2 **Heterologous expression of *aiiA *lactonases affects production of exoenzymes and secondary metabolites by *S. plymuthica *G3**. Table showing the transconjugant strain G3/pME6863 reduced chitinolytic (48 h) and proteolytic (48 h) activities which played a key role in biocontrol activity, indicated by the smaller halo diameter, compared to the control G3/pME6000 and the wild type. However the biosynthetic level of auxin indole-3-acetic acid (IAA) was five times higher in G3/pME6863 (2.77 ± 0.01 μg/ml) than in the control strain G3/pME6000 (0.57 ± 0.01 μg/ml) using HPLC analysis, when grown in LB supplemented with tryptophan for 48 h at 30°C. Siderophore production measured at 36 h was AHL-independent. Click here for file
