# Supplementary material for: Characterisation of two quorum sensing systems in the endophytic Serratia plymuthica strain G3: differential control of motility and biofilm formation according to life-style
Source: BMC Microbiol. 2011 Feb 1;11:26. doi: 10.1186/1471-2180-11-26 (PMC3044098; doi:10.1186/1471-2180-11-26)
Supplement: Additional file 2 — Heterologous expression of aiiA lactonases affects production of exoenzymes and secondary metabolites by S. plymuthica G3. Table showing the transconjugant strain G3/pME6863 reduced chitinolytic (48 h) and proteolytic (48 h) activities which played a key role in biocontrol activity, indicated by the smaller halo diameter, compared to the control G3/pME6000 and the wild type. However the biosynthetic level of auxin indole-3-acetic acid (IAA) was five times higher in G3/pME6863 (2.77 ± 0.01 μg/ml) than in the control strain G3/pME6000 (0.57 ± 0.01 μg/ml) using HPLC analysis, when grown in LB supplemented with tryptophan for 48 h at 30°C. Siderophore production measured at 36 h was AHL-independent. [file 1471-2180-11-26-S2.DOC]

| Phenotype G3 WT G3/pME6863-*aiiA* G3/pME6000 |
| --- |
| Chitinase (mm) 9.67±0.29 7.58±0.38 9.58±0.38  Protease (mm) 15.17±0.28 8.08±0.52 15.00±0.00  Siderophores (mm) 21.94±0.47 22.01±1.23 22.32 ±0.86  IAA production (μg/ml) 0.54±0.01 2.77±0.01 0.57 ±0.00 |
