# Supplementary material for: Characterisation of two quorum sensing systems in the endophytic Serratia plymuthica strain G3: differential control of motility and biofilm formation according to life-style
Source: BMC Microbiol. 2011 Feb 1;11:26. doi: 10.1186/1471-2180-11-26 (PMC3044098; doi:10.1186/1471-2180-11-26)
Supplement: Supplementary file 2 [file 1471-2180-11-26-S1.DOC]

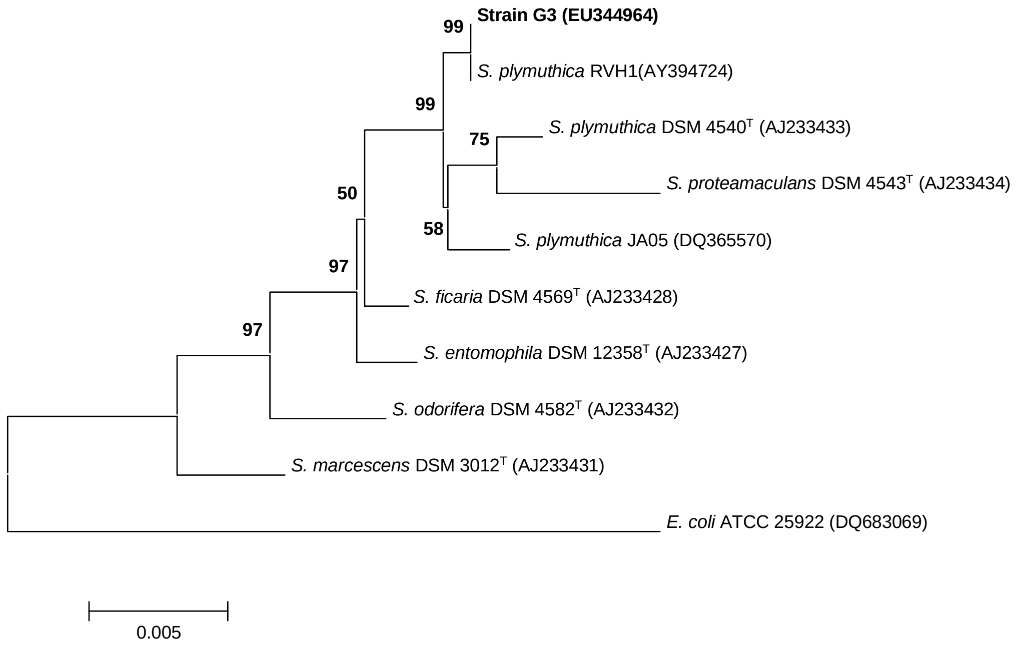
Strain G3 (EU344964)
S. plymuthica RVH1(AY394724)
S. plymuthica DSM 4540T (AJ233433)
S. proteamaculans DSM 4543T (AJ233434)
S. plymuthica JA05 (DQ365570)
S. ficaria DSM 4569T (AJ233428)
S. entomophila DSM 12358T (AJ233427)
S. odorifera DSM 4582T (AJ233432)
S. marcescens DSM 3012T (AJ233431)
E. coli ATCC 25922 (DQ683069)
99
99
75
50
58
97
97
0.005
